# Spatial analysis and associated factors of underweight, overweight and obesity among non-pregnant women of reproductive age in Zambia

**DOI:** 10.4314/ahs.v24i3.33

**Published:** 2024-09

**Authors:** Samson Shumba, Choolwe Jacobs, Mwila Kapembwa, Thomas Osman Miyoba, Nedah Chikondi Musonda

**Affiliations:** University of Zambia - Ridgeway Campus, Department of Epidemiology & Biostatistics, School of Public Health

**Keywords:** Underweight, overweight, obesity

## Abstract

**Introduction:**

Malnutrition in women in reproductive age (WRA) is associated with distinct types of public health problems such as preterm birth, low birth weight, malnourished children, poor psychological health and high mortality. The study aimed at investigating the risk factors associated with underweight, overweight and obesity among WRA and map their spatial distribution.

**Methods:**

The study used the 2013/2014 Zambia Demographic Health Survey (ZDHS) data and survey proportional odds model was fitted to the data. Spatial effects were modeled using Quantum Geographic Information System (QGIS) version 2.18.1 to develop univariate choropleth maps.

**Results:**

14,377 WRA were enrolled into the study. Overweight was more prevalent (15.87%) compared to underweight (10.22%) and obesity (6.47%). The findings revealed that a higher wealth index, age, having a car, using contraceptives, being married or divorced similarly increased the odds of being at or beyond a particular category of nutrition status. The spatial analysis also suggested Lusaka and Copperbelt were more prone to overweight and obesity, whereas Western had increased prevalence of underweight.

**Conclusion:**

Appropriate nutritional and economic programs are highly recommended in specific provinces of Zambia. Programs to curb overweight and obesity should be directed more to Lusaka and Copperbelt while underweight programs to Western province.

## Background

Overweight and obesity are leading risk factors of global death and disability and are often linked with various non-communicable diseases, such as hypertension, diabetes, cancer and cardiovascular disorders 1, 2. Globally, about one-third of adults are overweight or obese and about 10% of adults are underweight 3. In 2016, about 2 billion adults were either overweight or obese whereas about 600 million were underweight 4. In Bangladesh, the overall prevalence of being underweight was 25.2% among theurban non-pregnant women, with other studies reporting a prevalence ranging from 29.3% to 44.0%^4^. In India, prevalence of underweight among women was reported at 38.5% 5.

Studies in Africa suggests a double burden of malnutrition (underweight, overweight/obesity) 6. Despite the high prevalence in underweight, Africa has continued to register increasing numbers of overweight and obesity more especially in the urban areas 7, 8, 9. The prevalence of overweight and obesity in sub-Saharan Africa range from 20 to 25% of the urban population 10. A study in Kenya from 2003 to 2009 suggests that obesity has been on the rise and more common in mothers and children 11. Over the course of 10 years, from 2005 to 2015, the prevalence of overweight and obesity significantly increased from 18.3% to 30.5% among women in Mozambique. Furthermore, overweight and obesity was found to be more prevalent in urban areas and among women aged 18 to 24 years 12. In Burkina Faso, the prevalence of overweight and obesity was 13.82% and 4.84% respectively 13. In Malawi the combined prevalence of overweight and obesity in adult non-pregnant women was estimated to be 23.1%, in Nigeria it was 29.2% 14 and in Uganda it was 11.3% 15.

Developing countries today are undergoing various transitions ranging from social, economic, epidemiologic to nutrition thus underweight, overweight and obesity have all emerged as prominent health paradox among other contentious health problems today 16. Biological and behavioral factors such as hormonal fluctuations and inadequate physical activity during childhood can result in differences between males and females in terms of their susceptibility to weight-related issues. As a result, females are more likely to experience conditions of being underweight, overweight and obese as compared to males^17^,^18^,^19^. These conditions are associated with distinct types of public health problems such as diabetes mellitus, cardiovascular disease, cancer, stroke, respiratory infections, high cholesterol, high blood pressure, asthma and arthritis whereas being underweight is associated with preterm birth, low birth weight, malnourished children, poor psychological health and high mortality. Most underweight cases emanate from developing countries. The prevalence of chronic under-nutrition among women in SSA is 10-20%, while the prevalence of acute under-nutrition is 20-25% 20. Maternal undernutrition is estimated to be responsible for about 20% of childhood stunting 21. Studies have shown that women in developing countries are more vulnerable to under-nutrition due to several factors such as limited access to food, health care and education when compared to men.

Studies in Africa suggest all levels of weight distribution^7^. Despite the high prevalence in underweight, Africa has continued to register increasing numbers of overweight and obesity more especially in the urban areas 8, 9, 10. The rates of overweight and obesity have been on the rise in Sub-Saharan Africa ranging from 20 to 25% of the urban population 11. A study in Kenya from 2003 to 2009 suggests that obesity has been on the rise and more common in mothers and children 12.

Zambia being a low income country is increasingly facing health challenges related to nutrition and transitions that impact body weight change. The country is experiencing a public inconsistency of both under and over nutrition. Malnutrition among women not only has a major impact on their own health, but also on their children. A chronically undernourished woman is likely to give birth to an undernourished child, causing the cycle of undernutrition to be repeated over generations 22.

The study aimed at exploring the spatial distribution of underweight, overweight and obesity in WRA as an integral part to designing interventions that target high-risk areas with geographic information systems (GIS) techniques and to investigate their geographic distribution.

## Methods

### Source of Data

The sampling frame used for the 2013/2014 ZDHS was the Census of Population and Housing (CPH) of the Republic of Zambia, conducted in 2010 by ZamStats. The 2013/2014 ZDHS followed a stratified two-stage sample design. The first stage involved the selection of sample points (clusters) consisting of enumeration areas (EAs). EAs were selected with a probability proportional to their size within each sampling stratum. The second stage involved systematic sampling of households. A household listing operation was undertaken in all of the selected clusters. An average number of 133 households were contained in each cluster, from which a fixed number of 25 households were selected through an equal probability systematic selection process, to obtain a total sample size of 13,625 households. Results from this sample are representative at the national, urban and rural, and provincial levels 23. The study further excluded women who were pregnant during the time of collection of data or reported, missing BMI category, infecund and sterilized. The University of Zambia Biomedical Research Committee, ref number 1758-2021 and the National Health Research Authority approved this study.

### Dependent and independent variables

The variable of interest in this study was nutrition status (underweight, overweight and obesity) of women of reproductive age group. Nutrition status/body mass index is a person's weight in Kilograms (pounds) divided by the square of height in meters (or feet), Kg/m^2^ 23. Weight was typically measured using a calibrated electronic weighing scale. Height was generally measured using a stadiometer, a device specifically designed to measure height 24. Women of reproductive age group according to WHO are women between the age of 15 and 49 years. Underweight is body mass index (BMI) less than 18.5 Kg/m^2^, overweight BMI ranges from 25.0 to 29.9 Kg/m2 and obesity 30.0 Kg/m^2^ and above 25. The explanatory variables that were used in the study were demographic, socio-economic, behavioral and community level factors.

### Data analysis

For descriptive purposes, frequencies and percentages were computed for categorical variables. To determine association between the outcome variable (BMI status) and the categorical variables, the Uncorrelated Design Based Chi-square test (Rao – Scott Chi-square test) was used. 16.17 (538/3,330) percent of underweight women were aged 15 to 25 years, compared to 8.22% and 1.02% for overweight and obese women respectively. Western province recorded 19.44% (172/883); the highest number of underweight women. Lusaka province recorded 8.25% (242/2933) the lowest underweight cases. On the other hand, Lusaka reported the highest percentage of overweight and obesity at 22.11% (1676/2933) and 12.49% (648/2933) respectively.

Rural areas recorded high prevalence of underweight (11.8%) and recorded a low prevalence of overweight (11.48%) and obese (2.79%). Urban areas recorded a high prevalence in overweight (20.82%) and obesity (10.62%). Furthermore, the prevalence of underweight was 15.37% among poorest households, 12.49% poorer households, 10.08% medium wealth household, 7.42% and 7.73% for richer and richest household respectively. Overweight and obesity was consistently high in households with higher wealth status. Furthermore, currently smoking, drinking alcohol, HIV positive status and using contraceptives was associated with a high prevalence of overweight (25.25%, 21.04%, 17.94% and 20.31% respectively).

**Table:** 

Models	Akaike Information Criteria (AIC)	Bayesian Information Criteria (BIC)	Log-likelihood	Brant Test
Accepted Model (1)	18057.227	18379.05	-8984.613	0.2007
Competing Model (2)	18058.802	18395.245	-8983.356	0.1379
Competing Model (3)	18058.712	18387.845	-8983.401	0.1215

Overall, the results in table 2 shows that only HIV status was not associated with BMI status (p=0.0765).

Multivariable Survey Proportional Odds Model (SPOM) The study investigated the factors associated with underweight, overweight and obesity (BMI status) using the survey proportional odds model. The findings suggest that women aged 20 to 49 had increased odds of being beyond a particular category of nutrition status compared to women aged 15 to 19 years, given the effects of all other predictors are held constant. In other words, women aged 20 to 49 years were associated with increased odds of being obese compared to women aged 15 to 19. In the same vain, women with secondary and tertiary education had increased odds of being beyond a particular nutrition category compared to women with no level of education (AOR, 1.53; 95% CI, 1.25 – 1.88; p<0.001; and AOR, 1.89; 95% CI, 1.42 – 2.52; p<0.001 respectively)). A higher wealth status among women in reproductive age group was associated with increased odds of being beyond a particular nutrition category compared to women with a poorest wealth status, holding all other factors constant (AOR (poorer), 1.62; p<0.001; AOR (middle), 1.62; p<0.001; AOR (richer), 2.76; p<0.001; AOR (richest), 3.19; p<0.001).

Women with a positive HIV status had reduced odds of being beyond a particular BMI status (obesity) compared to women reported HIV negative, holding all factors constant (AOR, 0.68; 95% CI, 0.58 – 0.80; p<0.001). In other words, HIV positive women had increased odds of being underweight compared to HIV negative women. Holding all factors constant, women in Eastern province (AOR, 1.39; 95% CI, 1.15 – 1.68; p=0.001), and Lusaka province (AOR, 1.24; 95% CI, 1.02 – 1.50; p=0.027) had increased odds of being overweight/obese compared to women in central province, see table 3. However, women in Western province had reduced odds of being at a higher nutrition category than Central Province, alternatively they had increased odds of being underweight compared to women in Central province (AOR, 0.65; 95% CI, 0.51 – 0.83; p=0.001).

Overall, demographic, socio-economic, behavioral and community level factors were predicting nutrition status among women in reproductive age group(P<0.05), see table 3.

### Spatial Distribution of Underweight Women in reproductive age (15-49 years) in Zambia

Western province reported more women in reproductive age group were underweight 19.44% with Muchinga province which reported 13.84%. However, North western, Lusaka and Eastern province had the least prevalence of underweight represented by 8.85%, 8.25% and 7.84% respectively (see figure 1).

**Figure:**
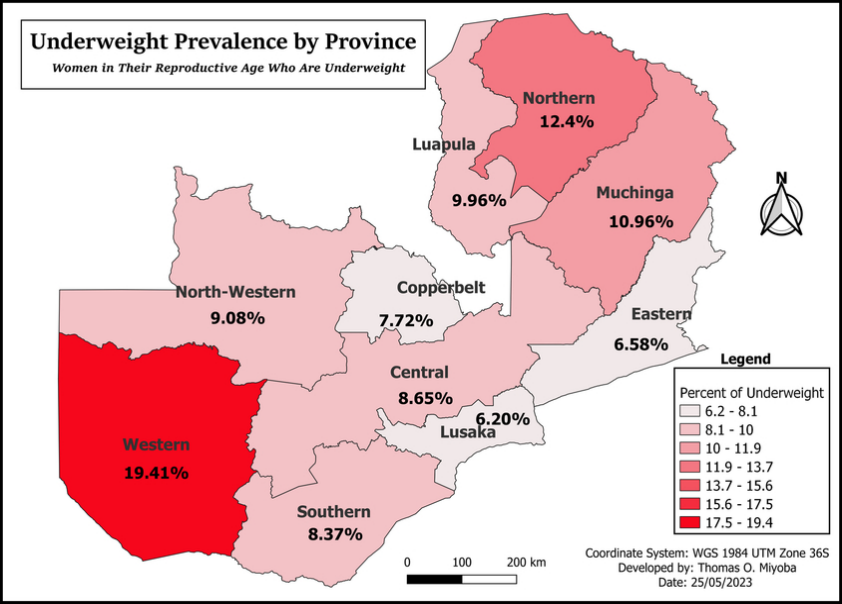
Underweight Prevalence by Province

### Spatial Distribution of Overweight Women reproductive age (15-49 years) in Zambia

The findings from 10 provinces in Zambia show that, Lusaka and Copperbelt province had the highest percentages of women in reproductive age group who were overweight, 22.11% and 19.86% respectively. Muchinga (10.33%), Northern (10.07%) and Western (8.61%) recorded the lowest proportion of overweight (see figure 2).

**Figure:**
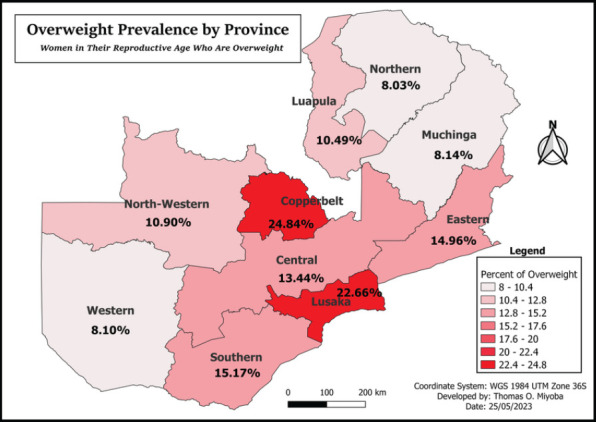
Overweight Prevalence by Province

### Spatial Distribution of Obesity Women reproductive age (15-49 years) in Zambia

In the study, Lusaka (12.49%) province had the percentage of reproductive women who were obese. Copperbelt province was second with 9.73% of obese cases in the province. Eastern, Muchinga, Northern and Western recorded the least proportion of obese women in reproductive age group were recorded in Western (see figure 3).

**Figure:**
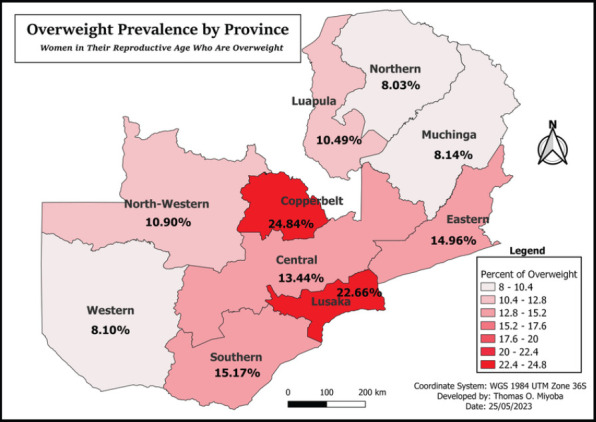
Overweight Prevalence by Province

## Discussion

The study aimed at investigating the spatial distribution and the risk factors associated with underweight, overweight and obesity among non-pregnant women of reproductive age in Zambia. This study used Zambia Demographic and Health Survey (ZDHS) data collected in 2018. The prevalence of underweight was 10.22%, 15.87% overweight and 6.47% obesity. The prevalence reported are similar to Uganda's, 12.4% overweight and 3.9% obesity 30. The prevalence of underweight in Egypt was reported 12.6%. The prevalence of underweight in Benin, Djibouti, Egypt, Ghana, Malawi, Mauritania and Morocco ranged from 12.6% to as high as 31.9%. Ghana had the least prevalence of overweight were as Egypt recorded the highest (31.4%). Obesity ranged from 0.6% in Benin to as high as 9.3% in Egypt 31. In Burkina Faso, the prevalence of overweight and obesity was 13.82% and 4.84% respectively 13. In Malawi the combined prevalence of overweight and obesity in adult non-pregnant women is 23.1%, 29.2% in Nigeria 14 and 11.3% in Uganda 15.

Developing countries across various regions, particularly in Africa, have consistently faced the challenges of under nutrition, alongside the emerging issue of over nutrition, resulting in a double burden of malnutrition 29. The rapid increase of overweight and obesity has been ascribed to changes in diet and lifestyle brought on by economic growth, rapid urbanization and globalization. In other societies in SSA, obesity is culturally accepted and in other cases desirable, particularly for women 32. The underlying cause of the rise in global overweight is frequently attributed to urbanization or substantial population growth in urban areas of low middle-income Countries 33.

The findings in this study confirms that underweight, overweight and obesity are a serious public health challenge. Studies have shown that obesity in women of reproductive age is associated with a high risk of infertility and gestational complications such as hypertensive disorders, gestational diabetes, hemorrhage and caesarean delivery, which have increased risk of fatal and infant death, neural tube defects and newborn macrosomia 34,36. Underweight on the other hand is linked with reduced fertility as well and adverse pregnancy complications including low birthweight, preterm birth and neonatal death 37, 38. Although Zambia still is greatly affected by epidemics of infectious diseases, especially HIV, the estimated national prevalence of overweight weight and obesity was 26.4% in 2010 39.

The study found that age, wealth index, province, type of residence (rural/urban), owning a car/truck, frequency of work, smoking cigarettes and HIV status were associated with underweight, overweight and obesity. The study further showed that 10.07%, 17.94% and 7.06% who were HIV positive were underweight, overweight and obese respectively. Similar to a study in South Africa found that more HIV infected women were overweight than underweight. 8.3% of HIV infected women were underweight and 17.2% and 3.6% were overweight and obese respectively 40. Smoking in the study was associated with nutrition status. However, WHO in 2006 associated HIV infection progression with weight loss 41. These findings are consistent with other studies 42-45 which relates smoking to decreased appetite and calorie intake and enhanced metabolism and reduced fat accumulation. This is a result of nicotine on the brains regulation of appetite and energy expenditure 45, 46.

Women from wealthy households or urbanized provinces in the study had a higher risk of being overweight or obese. The results of this study are consistent with previous studies from South Asia and sub-Saharan Africa countries, which revealed that women from wealthy and urbanized locations were at higher risk of overweight and obesity 47. The probabe reason for the relationship between urbanized provinces or areas and overweight and obesity could be that women who resided in households from urbanized areas have a reduced level of economic stress, physical activity and less healthy dietary habit (such as poor consumption of fruits and vegetables and a higher intake of highly caloric foods) compared to those from less urbanized areas or provinces 29.

Lusaka, Copperbelt, Eastern and Southern province (prominent urban regions in the country), registered high numbers of overweight and obese cases compared to other provinces. This coincides with the findings in other studies that the prevalence of overweight in developing countries was high ranging from 10.3% to 69.9%^31^. Urbanization has been known to be associated with refined sugars and animal fats, mostly coupled with sedentary lifestyle; all these are known to cause overweight or obesity 10. On the other hand, Western, Luapula and Muchinga province recorded a high prevalence of underweight. Similarly, these three provinces registered low cases of overweight and obesity. It is not unusual that the cases where more predominantly rural. In Ethiopia, poor regions showed an increased prevalence of underweight; similarly, the study has shown that well developed urban areas had high cases of overweight and obese 25. Furthermore, overweight and obesity was found to be prevalent in urban areas and among women aged 18 to 24 years 12.

The study also revealed that non-pregnant reproductive age women who resided in rural areas were less likely to be overweight or obese compared to those who resided in urban area. Women from rural provinces may be engaged in occupational physical activities (manual work) which promotes weight loss and less excess weight gain 26. Furthermore, the reduced intake of processed, packed and refrigerated foods by rural women could be the possible explanation for the negative relationship between rural province and overweight or obesity. The fndings of this study suggest that interventions to reduce underweight or undernutrition should be targeted on Western, Muchinga and Luapula province.

## Strengths and Limitations of the study

The strength of this study was in the use of national data, which is representative of the national population of non-pregnant women of reproductive age. Thus, the findings of this study are generalizable to the target population (non-pregnant women of reproductive age) in Zambia. The study also used spatial analysis to investigate the distribution of nutrition status (underweight, overweight and obesity); this independent analysis gives an additional support to the other statistical analysis in this study. However, it should be noted that the current study has its limitations, the latest ZDHS dataset (2018) did not capture weight and height of women therefore made it impossible to compute BMI which was the response variable in this study. Given that situated we were left to use the 2013/2014 ZDHS dataset. Secondly, the location of cases where only accurate to provinces, therefore spatial analysis was done at provincial level. Other variables fundamental to understanding underweight, overweight and obesity such as dietary intake, physical activity and sedentary lifestyle were not present in the ZDHS 2018 data set.

## Conclusion

In order to prevent or reduce the double burden of malnutrition (DBM) that is the coexistence of undernutrition or underweight along with overweight, obesity or diet related Non-Communicable Diseases within individuals, households and populations, interventions should target on the following provinces; Lusaka, Copperbelt, Eastern, Southern, Western, Luapula and Muchinga province. Additionally, interventional studies that evaluate the current policy initiatives in addressing underweight, overweight and obesity should be key priorities to improve reproductive women's health outcomes.
